# Retroclavicular vs Infraclavicular block for brachial plexus anesthesia: a multi-centric randomized trial

**DOI:** 10.1186/s12871-019-0868-6

**Published:** 2019-10-27

**Authors:** Andrés Felipe Gil Blanco, Pascal Laferrière-Langlois, David Jessop, Frédérick D’Aragon, Yanick Sansoucy, Natalie Albert, Pascal Tétreault, Pablo Echave

**Affiliations:** 1 Department of Anesthesiology, Medicine and Health Sciences Faculty, Centre Hospitalier Universitaire de Sherbrooke (CHUS), 3001, 12e Avenue Nord, Sherbrooke, QC J1H 5N4 Canada; 2 Department of Anesthesiology, Medicine and Health Sciences Faculty, Centre Hospitalier Universitaire de Laval (CHUL), 2705 boul Laurier, Quebec, G1V 4G2 QC Canada; 3 Department of Biomedical Engineering, Faculty of medicine and Dentistry, University of Alberta, 1098 Research Transition Facility, Edmonton, T6G 2V2 AB Canada

**Keywords:** Regional anesthesia, Brachial plexus, Upper extremity, Ambulatory, Anatomy

## Abstract

**Background:**

The coracoid approach is a simple method to perform ultrasound-guided brachial plexus regional anesthesia (RA) but its simplicity is counterbalanced by a difficult needle visualization. We hypothesized that the retroclavicular (RCB) approach is not longer to perform when compared to the coracoid (ICB) approach, and improves needle visualization.

**Methods:**

This randomized, controlled, non-inferiority trial conducted in two hospitals, included patients undergoing distal upper limb surgery. Patients were randomly assigned to a brachial plexus block (ICB or RCB). The primary outcome was performance time (sum of visualization and needling time), and was analyzed with a non-inferiority test of averages. Depth of sensory and motor blockade, surgical success, total anesthesia time, needle visualization, number of needle passes and complications were also evaluated. Subgroup analysis restricted to patients with higher body mass index was completed.

**Results:**

We included 109 patients between September 2016 and May 2017. Mean RCB performance time was 4.8 ± 2.0 min while ICB was 5.2 ± 2.3 min (*p* = 0.06) with a 95% CI reaching up to 5.8% longer. RCB conferred an ultrasound-needle angle closer to 0**°** and significantly improved needle visibility after the clavicle was cleared and before local anesthetic administration. No differences were found in the secondary outcomes. Similar results were found in the subgroup analysis.

**Conclusion:**

RCB approach for brachial plexus anesthesia was similar to ICB approach in terms of time performance. Needle visibility, which represent an important clinical variable, was superior and angle between needle and ultrasound probe was close to 0° in the RCB group.

**Clinical trial registration:**

ClinicalTrials.gov (NCT02913625), registered 26 September 2016.

## Background

Regional anesthesia (RA) offers several advantages over general anesthesia (GA) for upper limb orthopedic surgery. One of the advantages is the improvement in postoperative pain, which leads to decreased use of postoperative opioids needs and reduces the recovery time for patients [1, 2]. The infraclavicular (ICB) (coracoid) approach for brachial plexus anesthesia is recognized for its facility and simplicity to perform. However, an inevitable pitfall of this method is the steep angle between the needle and the ultrasound probe that makes needle visibility challenging [3–8]. Because of its different needle entry point, the retroclavicular (RCB) approach [9] offers an almost perpendicular needle-ultrasound (US) beam angle, but aims at the same goal as the ICB. This simple yet critical difference can theoretically improve block performance conditions [6]. Our group and others have provided evidence that this technique is efficient, rapid, safe and simple to perform [10–12].

The purpose of this randomized controlled trial was to perform a systematic comparison between these two RA techniques for upper limb surgery. By providing better needle visibility, our main hypothesis is that the total time required to perform RCB will be non-inferior to the time required to perform ICB while providing at least identical block quality and success. To that extent, several secondary outcomes were collected and analyzed such as needle visibility, imaging and needling time, depth of sensory and motor blocks, total anesthesia time, block success, angle and number of passes, procedural pain and incidence of complications.

## Methods

### Design and setting

We conducted this non-inferiority randomized trial at Centre hospitalier universitaire de Sherbrooke (CHUS, performing over 200 RCB per year) and Centre Hospitalier de l’Université Laval (CHUL, where this method was recently implemented), Quebec, Canada between September 2016 and May 2017. The trial was approved by the research ethics board of *Centre intégré universitaire de santé et service sociaux de l’Estrie (*MP-31-2017-1298), which provided a provincial-wide approval. Clinical Trial Registration: ClinicalTrials.gov (NCT02913625). All study participants provided written informed consent before randomization. A detailed study protocol was previously published [10, 13]. This study fully adhere to CONSORT guidelines [14].

### Study participants

All patients scheduled for elective or urgent surgery (mainly open reduction and internal fixation of arm and forearm, fasciectomy, epicondylitis, elbow and wrist arthroscopy and elbow arthroplasty) were eligible for the study if they were ≥ 18 years old; ASA (American Society of Anesthesiologists) class I-III; able to provide a valid written consent and weighed > 50 kg, regardless of body mass index (BMI). Patients with previous surgery or gross anatomical deformities of the clavicle, systemic or local infection at the needle entry point, coagulopathy, severe pulmonary disease, local anesthetic (LA) allergy, known neuropathy affecting the operated limb, pregnancy and for whom surgeon requested an indwelling catheter for postoperative analgesia were excluded.

### Intervention

In the intervention group, an RCB was performed by inserting the needle at the supraclavicular fossa and aiming the posterior wall of the axillary artery, strictly in-plane with the US probe resting in the delto-pectoral groove [10]. For the control group, the ICB was performed by placing the US probe in the infraclavicular fossa, medially to the coracoid process and by directing the needle towards the posterior wall of the axillary artery. Local anesthetic bolus (20 ml of 0.5% ropivacaine and 20 ml of 1.5% mepivacaine) was deposited under the axillary artery for both approaches. As defined a priori, ICB and RCB feasibility needed to be confirmed by a pre-scan before the randomization envelope was opened, to avoid recruitment of patients with gross anatomical deformation. Objective evaluation of motor and sensory blockade was required for all patients.

### Randomization and blinding process

Clinical and epidemiological research unit at the CHUS generated the random allocation sequence. Participants were randomly assigned to control or experimental group with a 1:1 allocation ratio and stratified by sites using permuted blocks of random sizes. To ensure concealment, block sizes will not be disclosed and sequentially numbered, opaque and sealed envelopes were used. Only the research assistant had access to the envelopes. A research assistant, resident or anesthesiology department staff member was responsible for assessing participant eligibility and recruitment. All anesthetic interventions were performed by an anesthesiology resident (minimally in its 2nd year of training) or an anesthesiologist of the recruiting centers. Before the study, all operators, including experienced attending anesthesiologists, had a minimum experience of three successful retroclavicular blocks, as well as a minimum overall experience of 20 regional blocks. Only the outcome assessor for the motor block and sensory block could be blinded since it was technically impossible to blind the person performing the block and the patient. To reinforce blinding of the motor and sensory block assessor, chlorhexidine and bandages were applied over the two theoretical needle entry points. Video assessors of needle visibility could not be blinded because needle position on the screen was evident, but the adjudication was made independently to minimize this bias.

### Outcomes assessment

A research assistant recorded the different times needed using a standard chronometer. Imaging time was the interval between initial contact of US probe with the skin and acquisition of a satisfactory axillary artery image. Needling time was the interval between the skin wheal and block needle withdrawal. Total anesthesia time was the sum of performance time (imaging plus needling) and the time required to achieve a sensory loss score of 9/10 (further described). The primary outcome, performance time, was measured in minutes (min) and corresponded to the sum of imaging time and needling time.

Detailed methodology of the following outcomes can be found in our previously published protocol [13]. Briefly, depth of sensitive and motor blocks was evaluated every 10 min after complete LA injection. Sensory loss was assessed in the territory of the radial, median, ulnar, musculocutaneous, and medial cutaneous nerve of the forearm using a 3-point score. As previously described [10, 15], the sum of these five scores represents the final sensory loss score (minimum score of 9/10 required). Motor function was tested for the radial, median, ulnar, and musculocutaneous nerves. The sum of these four scores represents the motor block final score [10, 15]. An independent, blinded, research assistant completed the sensory and the motor assessment and noted the final scores up to 30 min after block was performed. Blocks were considered successful only if surgery was completed without additional LA infiltration or GA. Blocks were recorded on video and two blinded independent anesthesiologists evaluated needle visibility using a 5-point Likert scale and ImageJ software (version 1.50i, developed by the National Institutes of Health [NIH]). This evaluation was done twice and needle angle on the horizontal axis was measured and noted before LA injection.

Immediately after complete LA bolus injection, an independent and blind outcome assessor evaluated procedural pain using a 10 cm visual analogue scale. Following our detailed protocol [13], number of needle passes (defined as positive integer unit each time the block needle needs to be realigned on the skin) as well as early and 48 h complications (such as paresthesia, vascular puncture, Horner syndrome, dyspnea, etc.) were also documented. We considered the use of neurostimulator as positive when the purpose of its use was not only for safety sentinel stimulation (< 0.3 mA). Details of complications and follow-up assessments are presented in our protocol [13]. Briefly, early complications were assessed on the day of surgery, all patients were further contacted 48 h after surgery to query for any delayed complications (dyspnea, paresthesia, weaknesses, pain, at the puncture site or hematoma). If a complication was suspected, patients were immediately referred for medical assessment.

### Statistical methods

The statistics software SPSS (version 24.0.0.0), SAS (version 9.4), R (version 3.5.1) and Graph Pad Prism (version 6.0 h) were used. Primary outcome was analyzed with the non-inferiority test of the averages. Data was not normally distributed and required a logarithmic transformation to perform pre-defined parametric tests. Primary outcome was interpreted based on the logarithmic transformation. Secondary outcomes were analyzed using superiority analysis. For continuous data or ordinal data with > 8 categories, data were compiled as averages and standard deviation. Student t test was used for parametric data and the Mann-Whitney test was used for non-parametric data. Chi square (data *n* > 5) or Fisher exact (data n < 5) test were used for dichotomous data. Finally, for ordinal parametric data the chi square test was used and the Mann-Whitney test was used for ordinal non-parametric data. Subgroup analysis were performed following the same criteria as the main group and was conducted to evaluate if BMI higher than our study population average was influencing the outcomes. For all analysis, the intention-to-treat principle was used for analysis of missing data. When required, results are reported as mean (SD). No interim analysis was performed.

### Sample size

Sample size calculation for this study has been presented in detail previously [13]. Briefly, it was calculated on the basis of a recent study [16] where the performance time for coracoid ICB approach was 5.6 min, with 45 s of visualization time and using our feasibility study recently conducted [10] where the needling time for the RCB approach was 3 min 42 s. As we explained in our published protocol [13] and based on our clinical judgement and statistical convention, we deemed that a time superiority of 5% would be significant and therefore have set the non-inferiority margin at 5%. An initial sample size of 49 patients per group was required to provide a statistical power of 0.9 (0.05 one-sided type 1 error), but to account for dropouts and inadequate procedures, the final sample size was set to 55 patients per group.

## Results

**Fig. 1:**
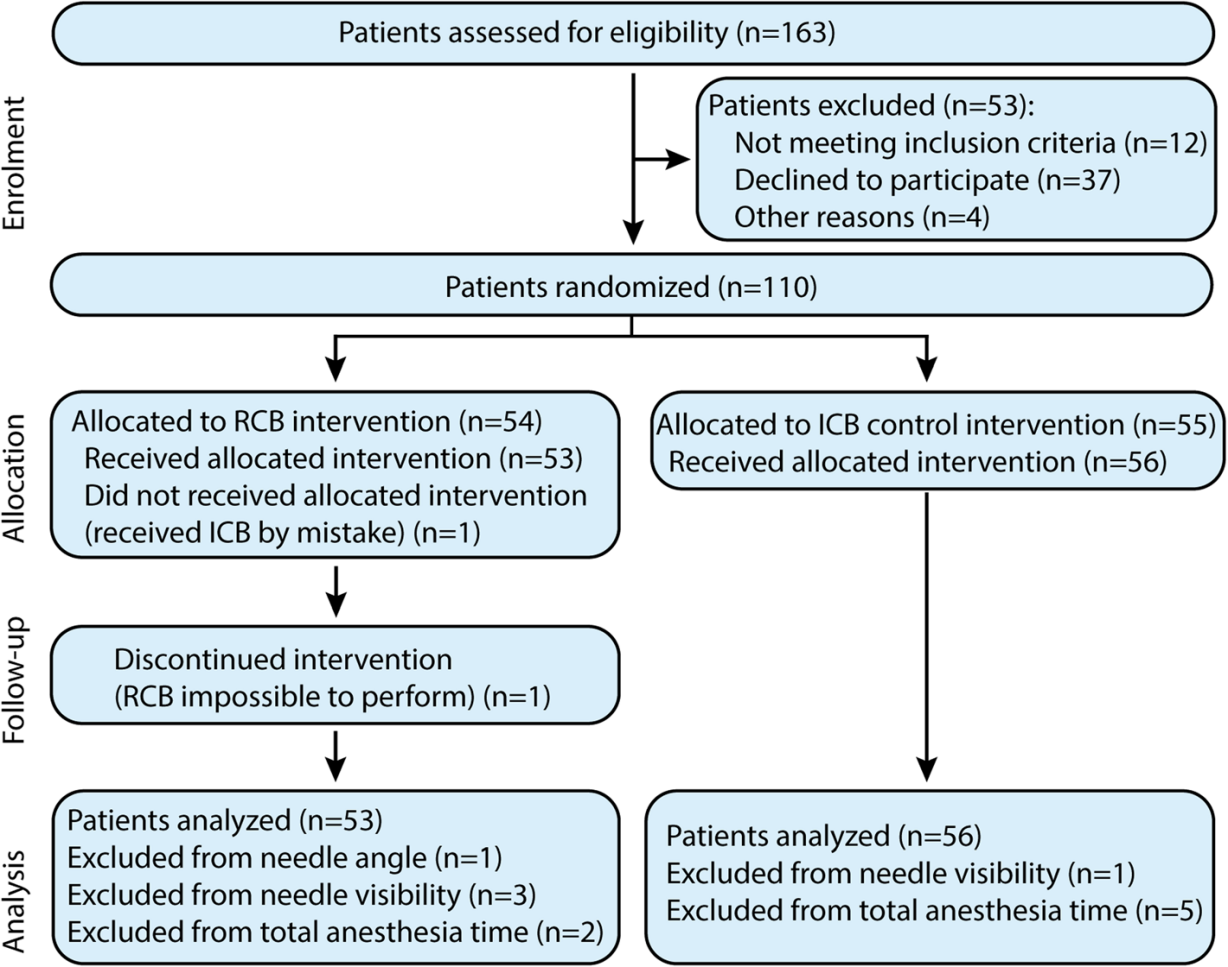
Study flow chart summarizes overall experimental design. A total of 163 patients were originally assessed for eligibility, from which 109 were analyzed. Grouping and dropout causes are indicated. Reason of exclusion for needle angle and visibility: improper image quality; Reason of exclusion for total anesthesia time: minimal sensitive block not obtained at 30 min. RCB, retroclavicular and ICB, infraclavicular approach

Between September 2016 and May 2017, 163 patients were screened, 110 patients were randomized in the trial, and 109 were available for analysis (Fig. 1). No patient was lost to follow up. Baseline characteristics are presented in Table 1.

**Table 1:** Demographics, ASA stratification and block repartition by center

	RCB (*n* = 53)	ICB (*n* = 56)
Age (years)	51 ± 16	52 ± 16
Female	16 (30%)	20 (36%)
BMI	26.6 ± 4.5	27.3 ± 5.1
ASA I	27 (51%)	22 (39%)
ASA II	24 (45%)	25 (45%)
ASA III	2 (4%)	9 (16%)
CHUL	38 (72%)	41 (73%)
CHUS	15 (28%)	15 (27%)

Data are given as number (%) or mean ± SD. Test of baseline characteristics were not performed [14]

*ASA* American Society of Anesthesiologists physical status classification score, *BMI* Body Mass Index, *CHUL* Centre Hospitalier Universitaire de l’Université Laval, *CHUS* Centre Hospitalier Universitaire de l’Université de Sherbrooke

### Primary outcome

**Fig. 2:**
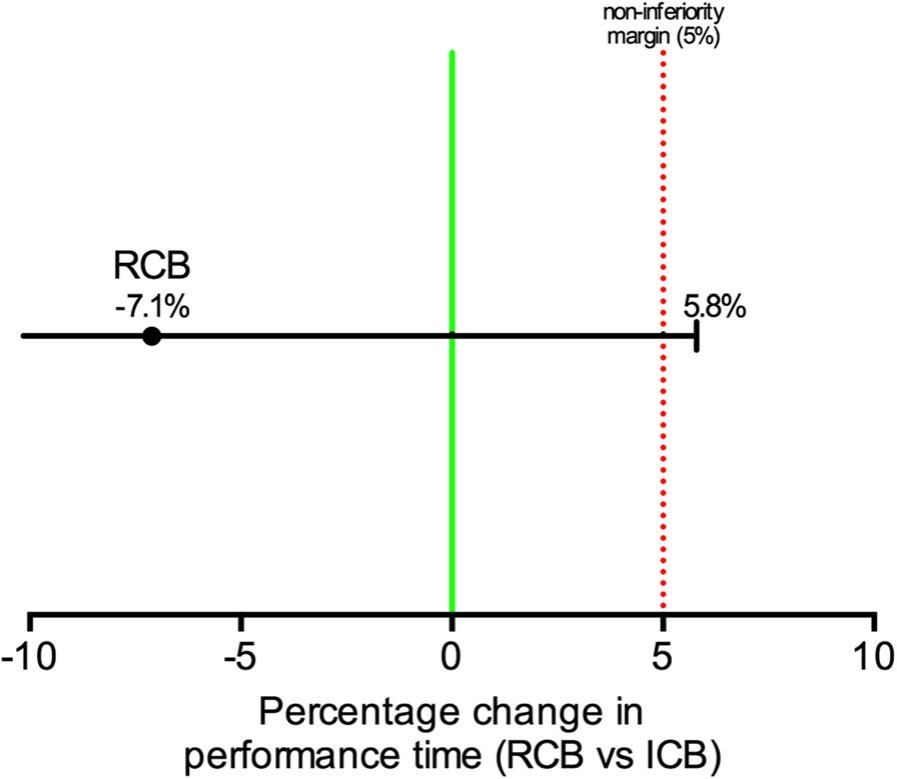
Visual representation of non-inferiority margin for the change in percentage of the performance time when comparing RCB approach to the standard ICB. The RCB approach is 7.1% faster than the ICB approach while the one-sided 95%CI is going over the 5% margin by 0.8%

The RCB group was composed of 53 participants and the ICB group was composed of 56. The average time required to perform an RCB was 4.8 min (SD 2.0 min) and 5.2 min (SD 2.3 min) for an ICB. Natural logarithmic average for performance time was 5.6 (SD: 0.4; 95% Confidence Interval [CI] 5.5–5.7) and 5.7 (SD: 0.4; 95% CI 5.6–5.8) for RCB and ICB respectively. Difference between the logarithmic averages was 0.073 (95% IC diff: [− 5.8% - infinity]; *p* = 0.06) or 7.1% when expressed in percentage change of performance time and was therefore going over the pre-specified non-inferiority margin of 5.0% by 0.8% (Fig. 2).

### Secondary outcomes

Imaging time did not show any differences between the two methods (35 ± 42 s for RCB vs 41 ± 52 s for ICB, *p* = 0.725). Similar results were observed for needling time, where no differences were present between groups (4.2 ± 1.7 min for RCB vs 4.5 ± 2.2 min for ICB, *p* = 0.702).

**Fig. 3:**
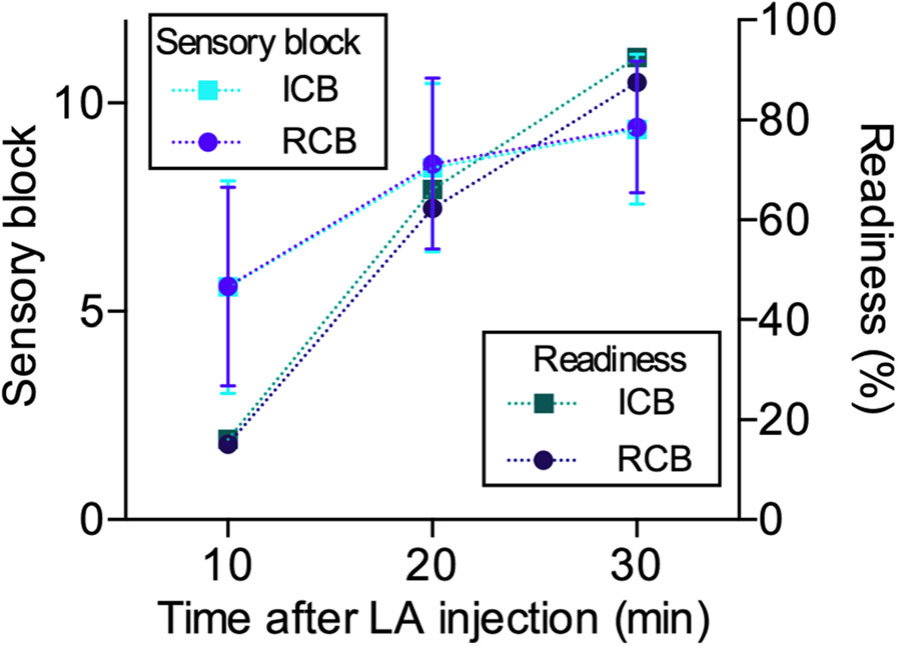
Sensory block performance and readiness for surgery did not show any differences between the two regional anesthesia approaches, providing a similar proportion of participants that could undergo their respective surgical procedure at each time points

A minimal sensory block score of 9/10 was also used to compute the proportion of patients that were ready for surgery as shown in Fig. 3. All individual scores are presented in Table 2. None of the time points showed significant differences between assessment of sensory loss (*p* = 0.71, 0.59 and 0.12 for 10, 20 and 30 min respectively), motor block (*p* = 0.75, 0.46 and 0.11 for 10, 20 and 30 min respectively) and readiness for surgery (*p* = 0.89, 0.68 and 0.39 for 10, 20 and 30 min respectively).

**Table 2:** Descriptive information about procedures and outcomes

	RCB	ICB	*p*-value
Performance time	4.8 ± 2.0 min	5.2 ± 2.3 min	*p* = 0.0608
Imaging time	35 ± 42 s	41 ± 52 s	*p* = 0.725
Needle time	4.2 ± 1.7 min	4.5 ± 2.2 min	*p* = 0.702
Total anesthesia time	*n* = 51	*n* = 51	
	27.3 ± 8.0 min	26.4 ± 8.3 min	*p* = 0.4331
Number of needle passes	1.1 ± 0.4	1.2 ± 0.7	*p* = 0.909
Neurostimulation use	37/53 (67.9%)	38/56 (69.8%)	*p* = 0.826
Needle angle	7.8 ± 6.4°	32.3 ± 10.3°	***p*** **< 0.001**
Procedural pain	2.9/10 ± 1.6	2.9/10 ± 1.9	*p* = 1
Needle visualization	*n* = 51	*n* = 55	
Likert evaluation time 1	3.49/5 ± 1.044	2.84/5 ± 0.9	***p*** **< 0.001**
Likert evaluation time 2	4.47/5 ± 0.66	3.09/5 ± 1.05	***p*** **< 0.001**
Person performing the block			
Residents	*n* = 41 (77.4%)	*n* = 43 (76.8%)	*p* = 1
Staff anesthesiologist	*n* = 12 (22.6%)	*n* = 13 (23.2%)	*p* = 0.818
Sensory loss at 10 min	5.6/10 ± 2.38	5.59/10 ± 2.55	*p* = 0.706
Sensory loss at 20 min	8.55/10 ± 2.05	8.45/10 ± 2.02	*p* = 0.587
Sensory loss at 30 min	9.43/10 ± 1.58	9.38/10 ± 1.80	*p* = 0.121
Ready for surgery at 10 min	*n* = 8 (15.1%)	*n* = 9 (16.1%)	*p* = 0.888
Ready for surgery at 20 min	*n* = 33 (62.3%)	*n* = 37 (66.1%)	*p* = 0.679
Ready for surgery at 30 min	*n* = 49 (87.5%)	*n* = 49 (92.5%)	*p* = 0.391
Motor block at 10 min	4.49/8 ± 1.77	4.25/8 ± 2.12	*p* = 0.750
Motor block at 20 min	6.70/8 ± 1.76	6.57/8 ± 1.99	*p* = 0.462
Motor block at 30 min	7.47/8 ± 1.50	7.13/8 ± 2.01	*p* = 0.112
Success of plexus block	50/53 (94.3%)	51/56 (91.1%)	*p* = 0.717
Sedation during surgery	*n* = 14	*n* = 23	*p* = 0.106
Rescue	*n* = 5	*n* = 7	*p* = 0.609
Local anesthesia	*n* = 2 (3.8%)	*n* = 3 (5.4%)	*p* = 1
Axillar block	*n* = 0	*n* = 3 (5.4%)	*p* = 0.244
Other block	*n* = 2 (3.8%)	*n* = 1 (1.8%)	*p* = 0.611
General anesthesia	n = 1 (1.9%)	*n* = 0	*p* = 0.486
Duration of surgery	49 ± 28 min	55 ± 29 min	*p* = 0.230
Immediate complications			
Paresthesia	*n* = 3 (5.7%)	*n* = 1 (1.8%)	*p* = 0.354
Arterial puncture	*n* = 1 (1.9%)	*n* = 4 (7.1%)	*p* = 0.364
Horner’s	*n* = 1 (1.9%)	*n* = 1 (1.8%)	*p* = 1
Late complications			
Pain at injection site	*n* = 2 (3.8%)	*n* = 4 (7.1%)	*p* = 0.679

Data are given as number (%) or mean ± SD. RCB indicates retroclavicular block; ICB, infraclavicular block; *p*-values in bold represent significant differences between RCB and ICB method

For 7 (6%) patients, the time needed to obtain a 9/10 sensitive score was longer than the time allotted for evaluation. Thus, participants with missing data were imputed. RCB total anesthesia time (*n* = 51) was 27.3 min (SD 8.0 min) and ICB total anesthesia time (*n* = 51) was 26.4 min (SD 8.3 min) (*p* = 0.433). Success of plexus block was similar in both groups: 50/53 (94.3%) for RCB and 51/56 (91.1%) for ICB (*p* = 0.717).

Images from 4 patients (one in ICB group and three in RCB group) were excluded because of poor acquired images. First Likert evaluation score was 3.49/5 (SD 1.04) for RCB and 2.84/5 (SD 0.90) for ICB (*p* < 0.001); and second Likert evaluation score was 4.47/5 (SD 0.66) for RCB and 3.09/5 (SD 1.05) for ICB (p < 0.001). Average angle to horizontal axis was 7.8° (SD 6.4°) for RCB (*n* = 52) and 32.3° (SD 10.3°) for ICB (*n* = 56) (p < 0.001).

Immediate complications consisted of three paresthesias (5.7%) in RCB group and one (1.8%) in ICB group (*p* = 0.35); one arterial puncture (1.9%) in RCB group and four (7.1%) in ICB (*p* = 0.364); and one Horner’s episode in each group (*p* = 1). Late complications consisted of two participants (3.8%) with pain at the injection site in RCB group and four (7.1%) in ICB group (*P* = 0.679); No significant difference was found between the groups. No patient developed permanent complications after follow-up (48 h).

### Subgroup analysis

Mean BMI was 27.13 kg/m^2^ (5.00 kg/m^2^) and patients with a higher than mean BMI were included in the subgroup analysis. Groups were composed of 27 participants for ICB and 24 for RCB (Table 3). Performance time for ICB was 6.10 min (SD 2.92 min) and for RCB was 4.99 min (SD 2.09 min) (*p* = 0.193) (Table 4). Poor quality images were imputed (analyzed data: RCB *n* = 22 and ICB *n* = 27). First Likert evaluation score was 3.45/5 (SD 0.76) for RCB and 2.85/5 (SD 1.03) for ICB (*p* < 0.05). Second evaluation score was 4.33/5 (SD 0.75) for RCB and 2.7/5 (SD 0.99) for ICB (*p* < 0.001). Needle angle was 8.48° (SD 7.14°) for RCB and 36.26° (SD 9.94°) for ICB (p < 0.001). Number of needle passes was 1.17 (SD 0.48) for RCB and 1.22 (SD 0.97) (*p* = 0.571).

**Table 3:** Subgroups demographics, ASA stratification and block repartition by center participants

	RCB (*n* = 24)	ICB (n = 27)
Age	46.6 ± 15.8	51.5 ± 12.4
Female	4 (16.7%)	9 (33.3%)
BMI	30.7 ± 3.7	31.2 ± 4.2
ASA I	13 (54.2%)	10 (37%)
ASA II	10 (41.7%)	14 (51.9%)
ASA III	1 (4.2%)	3 (11.1%)
CHUL	19 (79.2%)	20 (74.1)
CHUS	5 (20.8%)	7 (25.9%)

Test of baseline characteristics were not performed. Data are given as number (%) or mean ± SD. *ASA* American Society of Anesthesiologists physical status classification score, *BMI* Body Mass Index, *CHUL* Centre Hospitalier Universitaire de l’Université Laval, *CHUS* Centre Hospitalier Universitaire de l’Université de Sherbrooke

**Table 4:** Descriptive information about procedures and outcomes for subgroups analysis

	RCB (*n* = 24)	ICB (*n* = 27)	*p*-value
Performance time	4.99 ± 2.09 min	6.10 ± 2.92 min	*p* = 0.193
Imaging time	33.04 ± 17.82 s.	51.37 ± 67.78 s.	*p* = 0.713
Needling time	4.44 ± 2.04 min	5.24 ± 2.62 min	*p* = 0.238
Total anesthesia time	28.95 ± 9.02 min	28.67 ± 8.36 min	*p* = 0.982
Number of passes	1.17 ± 0.48	1.22 ± 0.97	*p* = 0.571
Needle angle	8.48 ± 7.14	36.26 ± 9.94	***p*** **< 0.001**
Visibility score	*n* = 22	*n* = 27	
Likert 1st evaluation	3.45/5 ± 0.80	2.85/5 ± 1.03	***p*** **< 0.05**
Likert 2nd evaluation	4.32/5 ± 0.78	2.70/5 ± 0.99	***p*** **< 0.001**

Patients above average BMI (27.13) were grouped according to the type of block they received. RCB indicates retro-clavicular block, *ICB*; infraclavicular block; *p*-values in bold represent significant differences between RCB and ICB method

## Discussion

For patients with a palpable supraclavicular fossa and no clavicle anatomical abnormalities, we were not able to prove complete statistical non-inferiority of the RCB in terms of performance time when compared to the ICB namely because both techniques were found to be efficient. However, when evaluating secondary outcomes, we found that total anesthesia time, needle and image time, blocks success and dynamics, complications incidence, procedural pain and number of needle passes were not different between approaches. These results therefore suggest that RCB is globally non-inferior when compared to ICB. Furthermore, these results were also found in the high BMI subgroup analysis.

Needle visibility is also an important clinical variable that can influence the choice of a method for brachial plexus anesthesia. As the needle approaches the plexus (Table 2, Likert evaluation time 2), RCB visibility score was > 1 point higher than ICB on Likert scale. This likely relates to the almost perpendicular angle (Table 2, needle angle) between US beam and needle. Good visibility provides a safer technique and a quicker needling time [6–8]. ICB and RCB actually confer an opposite gradient of visibility to sensitive structures. Improved needle visibility with RCB occurs near the neurovascular bundle as opposed to the limited visibility offered by ICB. The opposite occurs at or near the skin.

A recent anatomical study of RCB [17] highlights the potential risk of suprascapular nerve injury as the needle passes under the clavicle. No patients presented permanent neurologic complications during this study nor over the ~ 1000 RCB performed at the CHUS in recent years (albeit all of them were not performed within a research setting where this data is specifically recorded). Early and late incidence of paresthesia were also not considered statistically different between study groups, RCB paresthesia incidence was of 5.7% and is lower than reported by literature for ICB [8, 15, 18, 19]. A higher than usual arterial puncture rate for ICB can be linked to a lower needle visibility score inherent of the ICB method and possibly the primary nature of the study, performance time, which could lead clinicians to “beat the clock”. Nonetheless, no complications were recorded at 48 h for both groups.

The high BMI subgroup analysis, comprising almost 50% of the total number of patients recruited, also suggests that needle visibility and needle angle improvements with RCB over ICB are still present and not affected by a higher BMI. Our clinical experience leads us to infer that if the supraclavicular fossa is palpable and depressible, RCB is likely to be feasible even with high BMI patients, until an arterial depth of 5 cm is reached. In a time where there is increased interest, controversies and discussion about the RCB method [19–22], our study represents one of the first that properly compare these two methods and confirms its safety and efficacy.

## Limitations and Bias

Even if the majority of the blocks (> 75%) were performed by trainees in this study, success rate was comparable to more experienced clinicians (92.9% for resident and 92.0% for experience clinicians). Both procedures were still performed within a reasonable average time, but it is, however, likely that smaller standard deviation would be observed with experienced anesthesiologist only and therefore non-inferiority of the primary outcome could be statistically proven. It is important to mention that the RCB approach was newly introduced in the center where most patients were recruited but still present standard deviations similar to the ICB method, suggesting that this new regional anesthesia method appears to offer a fast learning curve. Likert scale was not validated previously in this context, but its use is becoming more popular [19, 23] and we deemed it appropriate to evaluate a secondary outcome of our trial. This is also one of the reasons we used performance time as our primary outcome even though needle visibility is clinically more important. We felt a pre-scan was necessary because needle travel under the clavicle can be impossible in case of unrepaired clavicle fracture. Pre-scan was done for both blocks and therefore did not limit internal validity although it did dilute imaging time. Despite these limitations, because randomization, blinding and adherence to protocol were strict and detection and experimental bias were limited we consider the internal validity to be good. We did not identify confounding factors.

## Conclusion

In the context of clinical reality where each approach possesses pros and cons, proving the usefulness of RCB is valuable as it offers another option to the regionalist. The implications of our work can be summarized as an improved strategy to easily keep track of the tip of the needle as it nears the neurovascular bundle. The downside of RCB is the acoustic shadow of the clavicle but our clinical experience so far leads us to believe that with strict in-plane US guidance, needle angled parallel to US probe and never posteriorly together with the confirmation of distance A and B [10] the RCB is a safe method. As previously observed [10], operators also found in this study that the fullness of the supraclavicular fossa and its lack of compressibility is increasing technical difficulty. Recent studies suggest that RCB could be used in emergency room settings because of its simplicity, safety and efficacity [24]. Taken altogether, despite falling short statistically to prove the non-inferiority in performance time, we consider that the absence of differences in all the other relevant secondary outcomes suggests that retroclavicular approach (RCB) represents an interesting alternative to coracoid approach.

## Data Availability

The dataset used and analyzed during the current study is available from the corresponding author on reasonable request.

## Abbreviations

ASA: American Society of Anesthesiologists
BMI: Body mass index
CHUL: Centre Hospitalier de l’Université Laval
CHUS: Centre hospitalier universitaire de Sherbrooke
GA: General anesthesia
ICB: coracoid
LA: Local anesthetic
NIH: National Institutes of Health
RA: Regional anesthesia
RCB: Retroclavicular
US: Ultrasound
